# A Hospital with Circular Wards

**Published:** 1884-08

**Authors:** 


### A Hospital with Circular Wards.

For some time past an attempt has been made to raise the funds needed to provide hospital accommodation for the town and district of Burnley, Lancashire. The plans approved of by the committee provide for the erection of a hospital with one story pavilion wards of the circular form, as suggested by Professor Marshall, President of the Royal College of Surgeons. Two of these wards are to be built at present, with an internal diameter of 60 feet, for the accommodation of twenty beds each.—*Ex.*

